# A Geomorphological Regionalization using the Upscaled DEM: the Beijing-Tianjin-Hebei Area, China Case Study

**DOI:** 10.1038/s41598-020-66993-9

**Published:** 2020-06-29

**Authors:** Bin Zhang, ZeMeng Fan, ZhengPing Du, JiLin Zheng, Jun Luo, NaNa Wang, Qing Wang

**Affiliations:** 1 State Key Laboratory of Resources and Environmental Information System, Institute of Geographic Sciences and Natural Resources Research, Chinese Academy of Sciences, Beijing, 100101 China; 2 Sichuan Provincial Engineering Laboratory of Monitoring and Controlling for Soil Erosion on Dry Valleys, China West Normal University, Nanchong, Sichuan 637009 China

**Keywords:** Geomorphology, Geomorphology

## Abstract

Characterizing geomorphological patterns based on digital elevation models (DEMs) has become a basic focus of current geomorphology. A new DEM upscaling method based on the high-accuracy surface modelling method (HASM-US method) has been developed to improve the accuracy of current models and the subjectivity of macroscopic geomorphological patterns. The topographic variables of elevation (EL), slope (SL), aspect (AS), relief amplitude (RA), surface incision (SI), surface roughness (SR), and profile curvature (PC) with a spatial resolution of 1 km × 1 km in the Beijing-Tianjin-Hebei (BTH) area of China have been obtained by using the HASM-US method combined with the principal component analysis (PCA) method in terms of the elevation data of the SRTM-4 DEM, meteorological station location information, and field measurements with a GPS receiver. A geomorphological regionalization pattern has been developed to quantitatively classify the geomorphological types in the BTH area by combining the seven topographic factors of EL, SL, AS, RA, SI, SR, and PC that have significant spatial variation. The results show that the upscaling accuracy of elevation (mean difference only −2.32 m) with the HASM-US method is higher than that with the bilinear interpolation method and nearest neighbour interpolation method. The geomorphologic distribution in the BTH area includes 11 types: low plain, low tableland, low hill, low basin, middle plain, middle hill, low mountain with low RA values, low mountain with medium RA values, middle mountain with low RA values, middle mountain with medium RA values, and middle mountain with high RA values. The low plain is the dominant geomorphological type that covers 40.58% of the whole BTH area. The geomorphological distribution shows the different significant characteristics: the elevation rapidly decreases from the Taihang Mountains to the eastern area, gradually decreases from the Yanshan Mountains to the southern area, and first increases and then decreases from the Bashang Plateau to the southeastern area in the whole BTH area.

## Introduction

Landform, one of the key components of geographical classification and regionalization, is the most important factor for identifying regional differences^1^. The types of landform are generally classified into three major categories of mountains, hills, and plains. Elevation and relative elevation differences as well as surface incision variations between these categories are obvious; regional relief is therefore the result of interactions between exogenic and endogenic forces that not only profoundly affect basic patterns and changes in other environmental factors but also directly influence regional land use as well as agriculture and industrial production.

Automatic classification of geomorphological types is currently based mainly on the use of regular statistical windows^2^, applying digital elevation models (DEMs) to extract a variety of surface morphological factors for classification, including elevation, slope, relief amplitude (*RA*), surface incision (*SI*), surface roughness (*SR*), profile curvature (*PC)*, and the elevation variance coefficient (*VC*)^3–11^. As topographic variables can, to some extent, reflect aspects of regional geomorphology, this approach forms the basis of geomorphological regionalization. This approach has been widely used in the field since Hammond^12^ first proposed that geomorphology can be classified on the basis of slope, relief, and profile types using statistical windows^1,13–15^. It remains the case, however, that topographic factors will not be exactly the same in all applications; Dragut^16^ used a suite of four parameters, elevation, profile and plan curvature, as well as slope gradient, while Liu utilized six parameters: relief amplitude, surface incision, surface roughness, and the elevation variance coefficient, as well as the mean slope and elevation^7^. In contrast, Hu^7^ selected just three parameters: relief amplitude, surface incision, and the terrain position parameter (*TPI*). Large-scale geomorphological patterns can mostly be extracted from SRTM DEM data^4,17,18^; indeed, a great deal of attention has been afforded to these data, as this application boasts near-global coverage (i.e., between 56°S and 60°N), has relatively high spatial resolution and is free of charge. SRTM DEM data outputs have therefore been applied widely^9,19^; in terms of regional-scale digital geomorphology, SRTM data have commonly been upscaled to obtain macroscopic geomorphological information^20^. The accuracy of DEM data is mainly controlled by the measurement accuracy, resolution of DEM data, the slope of a pixel, and modelling error of DEM model^20^, which depends to a large extent on sample data reliability^21^ and therefore means that simple resampling methods can significantly increase errors^19^. The traditional DEM models in this area suffers from a series of inherent shortcomings, including subjective parameter selection for depicting macrogeographical patterns as well as a greater level of error and the loss of detailed information from DEM data subsequent to upscaling processing. Therefore, on the same condition of the measurement accuracy, terrain complex level and resolution of DEM data, how to develop a suitable upscaling method to improve the DEM modelling accuracy is important in the geomorphological Regionalization.

**Figure 1:**
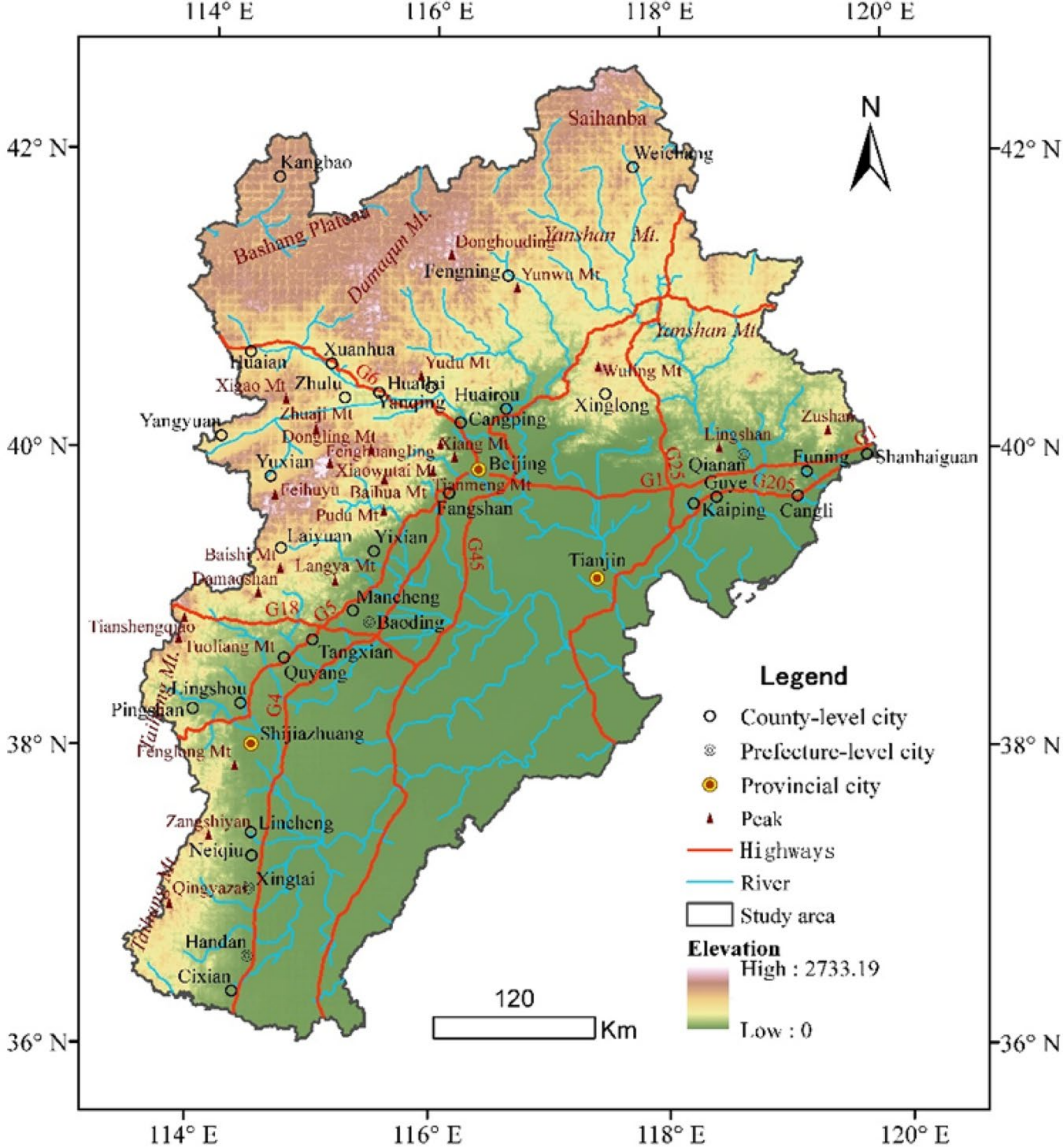
The study area in the BTH region.

The Beijing-Tianjin-Hebei (BTH) region is located between 113°04′-119°53′E and 36°01′-42°37′N within China (Fig. 1). Administrative zoning in this area includes the municipalities of Beijing and Tianjin as well as Hebei Province, encompasses a total area of 21.60 × 10^4^ km^2 ^^22^ and is the political, cultural, and economic centre of China. This region also includes the Taihang and Yanshan Mountains as well as the North China Plain, is located within the transitional zone between the second and third steps and thus contains both complex and diverse geological structures and types. The BTH region, therefore, has important political and economic status and occupies a very important physical geographical region within China. There has also been no research to date that specifically addresses the macroscopic geomorphological features of this area. The main objective of this paper is therefore to propose a new upscaling method and to reveal the basic characteristics and spatial patterns of geomorphology within the BTH region. These results not only reveal the macroscopic landform features of this region but also provide a scientific basis for the sustainable utilization of regional resources, urban and rural development, and industrial and agricultural layout.

## Data and Methods

### Data sources

The data sources used in this study include three categories. The first was SRTM-4 grid data (CGIAR-CSI, http://srtm.csi.cgiar.org/) with a spatial resolution of 3 arcsec (approx. 90 m^2^ at the Equator); multiple sets of these data were downloaded and projection transformed (as in the continuous operation reference station system, CORS). The second category of data comprised coordinate data from 102 meteorological stations at elevations less than 50 m within the BTH region but with large positional error eliminated. The third category comprised the elevation data of 458 field points with information on altitude and geographic coordinates observed using GPS-based from CORS.

### HASM-US method

A method for high accuracy surface modelling (HASM)^23–26^ has been developed since 1986 to integrate the extrinsic and intrinsic properties and find solutions for the error and multi-scale problems that are prevalent when each type of information is used separately^27^. This approach uses these inputs as its drivers as well as locally accurate information (e.g., ground observation and/or sampling data) as optimum control constraints. As this modelling approach is based on differential geometry and optimal control theories, it has been widely applied across numerous fields, including in Earth surface modelling DEMs^19,26–28^.

Applying the HASM algorithm, a HASM-upscaling (HASM-US) algorithm for DEM upscaling simulation was developed in this study.

Grids with different spatial resolutions within the same region, $\varOmega (H)$ and $\varOmega (h)$, are expressed as follows:

1 $$\varOmega (H)=\{(I,J)|(I\times H,J\times H)\},(0 < I\le {I}_{H},0 < J\le {J}_{H})$$

2 $$\varOmega (h)=\{(i,j)|(i\times h,j\times h)\},(0 < i\le {I}_{h},0 < j\le {J}_{h})$$

The spatial resolutions of these two grids are expressed as $H$ and $h$, where $H > h$, $H\times {I}_{H}=h\times {I}_{h}$, and $H\times {J}_{H}=h\times {J}_{h}$. Similarly, ${I}_{h}$ and ${J}_{h}$ denote the number of rows and columns in the coarse grid, respectively, while ${I}_{h}$ and ${J}_{h}$ denote the number of rows and columns in the fine grid, respectively. A digital terrain model (DTM) corresponding to this grid is $\{{f}_{h}\}=\{{f}_{i,j}=f(i,j),0 < i\le {I}_{h},0 < j\le {J}_{h}\}$, while the DTM corresponding to the grid is $\{{f}_{h}\}=\{{f}_{i,j}=f(i,j),0 < i\le {I}_{h},0 < j\le {J}_{h}\}$_._

Thus, assuming the grid resolution is .., the sample set is П, and the HASM algorithm based on the equality constraint is $HASM({\prod },h)$, as follows:

3 $$\{\begin{array}{c}min{\Vert {A}_{H}{X}_{H}^{(n+1)}-{b}_{H}^{(n)}\Vert }_{2}\\ s\cdot t\,{C}_{H}{X}_{H}^{(n+1)}={d}_{H}^{(n)}|\varPi \end{array}$$

where ${A}_{H}$ denotes the coefficient matrix of grid $\varOmega (H)$, ${X}_{H}^{(n+1)}$ is the unknown parameter, ${b}_{H}^{(n)}$ is the right item, ${C}_{H}$ is the coefficient matrix of the optimum control equation, and ${d}_{H}^{(n)}$ is the right end item of the optimal control equation.

**Figure 2:**
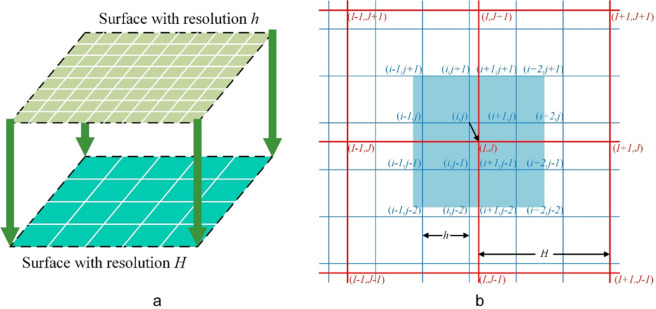
The Schematic diagram (**a**) and grid projection (**b)** of spatial upscaling in the HASM-US method.

Building on DTM grid data, $\{{f}_{H}\}$ comprises spatial resolution $h$ and point data П, while grid data $\{{f}_{H}\}$ of coarse spatial resolution $H$ was generated (Fig. 2(a)). Thus, during the upscaling process, a coarse-resolution DTM retains its overall trend and encompasses additional details by optimizing the control point data.

A projection was formed between coarse and fine grids throughout the process of spatial grid data upscaling. The coarse grid comprises the following 16 fine grids (Fig. 2(b)):

$$\begin{array}{c}(i-1,j+1),(i-1,j),(i-1,j-1),(i-1,j-2),(i,j+1),(i,j),(i,j-1),\\ \,(i-1,j-2),(i+1,j+1),(i+1,j),(i+1,j-1),(i+1,j-2),(i+2,j+1),\\ \,(i+2,j),(i+2,j-1),(i+2,j-2)\end{array}$$

The fine grid can therefore be simplified as follows:

4 $$\begin{array}{ccc}{\bar{X}}_{H}(I,J) & = & ({X}_{h}(i-1,j+1)+{X}_{h}(i-1,j)+{X}_{h}(i-1,j-1)+{X}_{h}(i-1,j-2)\\  &  & +{X}_{h}(i,j+1)+{X}_{h}(i,j)\\  &  & +{X}_{h}(i,j-1)+{X}_{h}(i,j-2)+{X}_{h}(i+1,j+1)+{X}_{h}(i+1,j)\\  &  & +{X}_{h}(i+1,j-1)+{X}_{h}(i+1,j-2)+{X}_{h}(i+2,j+1)+{X}_{h}(i+2,j)\\  &  & +{X}_{h}(i+2,j-1)+{X}_{h}(i+2,j-2))/16\end{array}$$

A simple arithmetic averaging method was then adopted to reduce computation, and a value of ${\bar{X}}_{H}(I,J)$ was obtained for more accurate simplification using the inverse distance interpolation method. The upscaling process of each coarse grid can therefore be similarly simplified; thicalculation process can be defined as a grid projection operator ${G}_{h}^{H}$, as follows: ${\bar{X}}_{H}(I,J)={G}_{h}^{H}({X}_{h})$.

As ${\bar{X}}_{H}$ is the trend surface, it is possible to calculate the residual of the sample point set П and the trend surface, which enables HASM simulation to be performed on residuals. A grid residual surface was therefore obtained by superimposing the trend surface, ${\bar{X}}_{H}(I,J)$, to obtain the final DTM. This upscaling algorithm was defined as HASM-US and was formulated as follows:

5 $$\{\begin{array}{c}min{\Vert {A}_{H}({X}_{H}^{(n+1)}-{\bar{X}}_{H})-{b}_{H}^{(n)}\Vert }_{2}\\ s.t\,{C}_{H}({X}_{H}^{(n+1)}-{\bar{X}}_{H})={d}_{H}^{(n)}|\varPi -{C}_{H}{\bar{X}}_{H}\end{array}$$

### Data processing

**Figure 3:**
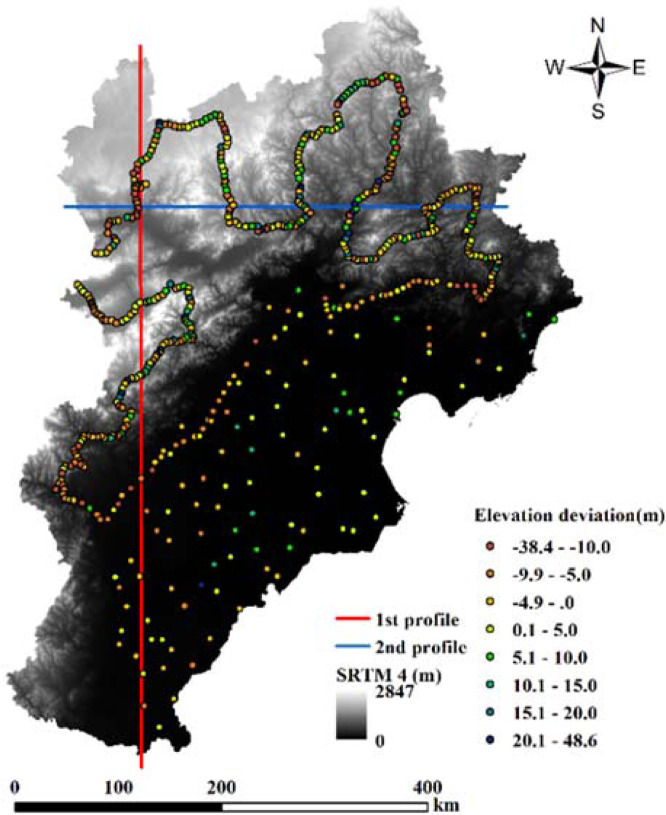
The locations of points and profiles across the BTH study area.

The ellipsoidal heights (*He*) derived from GPS measurements were converted to orthometric heights (*Ho*) of EGM96 used in the SRTM: *Ho = He – N*. Here, *N* is the geoid height. During the upscaling simulation process of SRTM data, the elevation value of measuring point by GPS instrument and meteorological station were used as the basic value, and the differences between the corresponding pixel value of SRTM data / upscaled DEM data and the basic value were calculated and used to validate the modelling errors of upscaled SRTM data with HASM-US and other methods. The elevation differences (Table 1) were calculated between 560 pixel values of SRTM sample data and the basic values, which correspond to 458 pixels of field measuring points and 102 pixels of meteorological stations, respectively. The calculated results show that the maximum positive difference and maximum negative difference in this analysis were 48.61 m and −38.41 m, respectively, encompassing a mean difference of 1.81 m, a root mean square (RMS) difference of 10.75 m, and a mean absolute difference of 7.65 m. The SRTM elevation values tend to be higher because measurement points are mainly distributed within the northern and western mountainous areas where *RA* is large; for this reason, an difference statistical SRTM analysis was performed using meteorological station elevation data. This additional analysis revealed that the overall difference is not large, especially within the flat plain area where the main meteorological stations are located. The data in Fig. 3 reveal the distribution of DEM grid value distributions at each sample point; within the northern and western mountainous areas of the BTH region, the SRTM data include relatively high elevation values, while relatively low elevation values are seen in the northeastern mountainous, central, and southeastern plains.

**Table 1:** Statistical characteristics of elevation difference (m).

Pixel type	Maximum positive difference	Maximum negative difference	Mean difference	RMS difference	Mean absolute value difference
Pixel of field observation point	48.6070	−38.4110	1.8140	10.7462	7.6533
Pixel of meteorological station	10.9000	−21.2000	−0.9029	5.6920	4.3520
All sample pixels	32.9509	−24.3160	1.3184	9.8730	7.0588

DEM data is one of the key parameters for many eco-environmental models. Except the DEM data, most eco-environmental models usually involve many other parameters such as vegetation, soil, climate and human activity data In fact, even though there is a high-resolution DEM data, if the high-resolution data of other parameters needed by the eco-environment model is very difficult to obtain, the high-resolution DEM data is often need to be upscaled to ensure union all parameters in the eco-environmental model^27^. Therefore, to overcome problems of missing detailed information and the loss of accuracy when directly resampling the DEM, SRTM grid data were scaled up using the high-accuracy surface modelling (HASM) method^19^. Combined with meteorological station coordinates and field-measured GPS data, DEM data for 1 km by 1 km cells were obtained; combined with meteorological station coordinates and field-measured GPS data, a series of parameters (i.e., elevation, slope, aspect, *RA*, *SI*, *SR*, profile curvature) were calculated using ArcGIS software. Applying the mean change-point analysis method^29^, it is possible to conclude that a grid size of 5 km by 5 km comprises the best window size for extracting BTH region *RA* from 1 km-resolution DEM data. Principal component analysis (PCA) was then used to screen dominant topographic factors to classify geomorphological types within the study area.

### Classification method of geomorphological types

**Figure 4:**
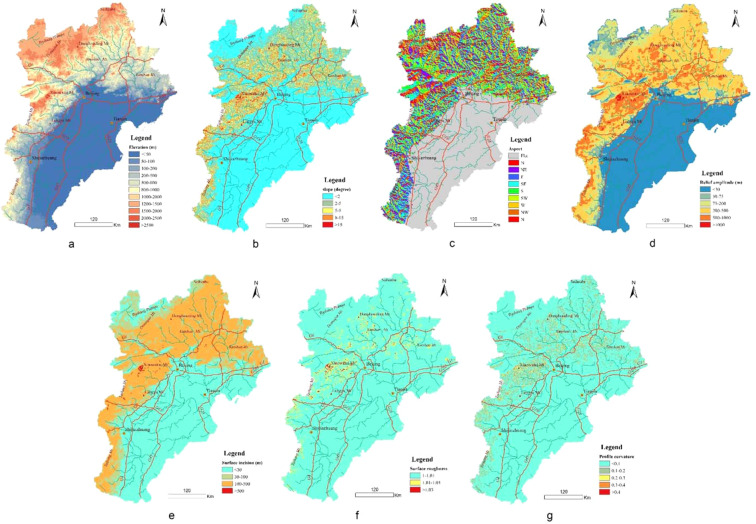
The spatial distribution of major geomorphological parameters in the BTH area (Notes: **a–g** express the distribution of topographic parameters of *EL*, *SL*,*SA*, *RA*,*SI*, *SR* and *PC*, respectively).

Terrain analysis is a prerequisite for geomorphological regionalization. Due to the complicated landforms in the study area, diverse common parameters should be selected to characterize the topographic features and geomorphological regionalization. According to Liu (2006)^30^, Hu (2015)^7^, and Li (2013)^31,9^, the parameters include elevation (*EL*), slope (*SL*), aspect (*SA*), relief amplitude (*RA)*, surface incision (*SI)*, surface roughness (*SR)* and profile curvature (*PC*) (Fig. 4).

Geomorphological studies carried out based on DEMs have led to a number of different correlations between parameters. Thus, to determine which parameters are most important, principal component analysis (PCA) was carried out (Tables 2–4). The cumulative contribution rate of the first two principal components (*Pc*1, *Pc*2) can reach up to 97.60%, while the highest correlation of the first Pc is elevation (0.950) and the second is *RA* (0.877). Elevation and *RA* were therefore used as parameters for classifying geomorphological types within the study area.

**Table 2:** Correlation coefficient matrix.

Parameters	*SA*	*EL*	*PC*	*SI*	*SL*	*SR*	*RA*
*SA*	1.00000						
*EL*	0.55094	1.00000					
*PC*	0.43258	0.45097	1.00000				
*SI*	0.47701	0.62241	0.74546	1.00000			
*SL*	0.43760	0.52359	0.70436	0.84529	1.00000		
*SR*	0.26242	0.35686	0.55462	0.69553	0.90421	1.00000	
*RA*	0.50615	0.58272	0.80864	0.96221	0.86020	0.69747	1.00000

**Table 3:** Eigenvalues and eigenvectors.

Parameters		Pc1	Pc2	Pc3	Pc4	Pc5	Pc6	Pc7
Eigenvalues		1.71E+05	1.92E+04	4.37E+03	2.94E+02	1.07E+00	4.84E-04	1.47E-06
*Eigenvectors*	*SA*	1.18E-01	1.26E-01	9.84E-01	−4.90E-02	4.89E-05	1.16E-05	3.57E-06
	*EL*	9.50E-01	−3.03E-01	−7.42E-02	1.82E-02	6.07E-05	−3.27E-08	7.30E-07
	*PC*	4.81E-05	1.70E-04	4.63E-07	3.23E-04	−1.20E-03	−1.00E+00	4.00E-03
	*SI*	1.21E-01	3.51E-01	−1.05E-01	−9.23E-01	6.48E-03	−2.40E-04	−4.12E-06
	*SL*	2.93E-03	8.21E-03	−1.62E-03	−2.95E-03	−1.00E+00	1.19E-03	−1.85E-03
	*SR*	3.26E-06	1.30E-05	−6.26E-06	−1.24E-05	−1.85E-03	4.00E-03	1.00E+00
	*RA*	2.63E-01	8.77E-01	−1.25E-01	3.82E-01	7.92E-03	2.75E-04	5.22E-06

**Table 4:** Variance PC contribution rates.

PC	Eigenvalue	Variance contribution rate (%)	Cumulative variance contribution rate (%)
Pc1	1.71E+05	87.73	87.73
Pc2	1.92E+04	9.87	97.60
Pc3	4.37E+03	2.24	99.85
Pc4	2.94E+02	0.15	100.00
Pc5	1.07E+00	0.00	100.00
Pc6	4.84E-04	0.00	100.00
Pc7	1.47E-06	0.00	100.00

The geomorphological regionalization scheme (Table 5) of the BTH area was approved by integrating with the actual situation, EL and RA parameters in the whole BTH area. The landform of the BTH area was classified into 11 geomorphological types: low plain, low tableland, low hill, low basin, middle plain, middle hill, low mountain with low RA values, low mountain with medium RA values, middle mountain with low RA values, middle mountain with medium RA values, and middle mountain with high RA values.

**Table 5:** The classification standard of geomorphological types in the BTH area.

Relief\ Elevation	Flat (<30 m)	Tableland (30 ~ 75 m)	Hill (75 ~ 200 m)	Low amplitude (200 ~ 500 m)	Medium amplitude (500 m ~ 1,000 m)	High amplitude (> 1,000 m)
Class 1 (<500 m)	Low plain	Low tableland	Low hill		—	—
Class 2 (500 ~1,000 m)	Low basin			Low mountain with low RA	Low mountain with medium RA	—
Class 3 (1,000 ~ 2,500 m)	Middle plain		Middle hill	Middle mountain with low RA	Middle mountain with medium RA	Middle mountain with high RA

(- indicates no such type).

## Results

### Accuracy validation

To verify the advantages and disadvantages of the upscaling algorithm used here combined with sample information and common resampling methods, the methods of cross-validation, bilinear interpolation, nearest neighbour interpolation, and HASM-US were all used to classify geomorphological types within the BTH study area. A total of 560 sampling points were randomly divided into ten components, of which nine (507 samples) were used as training samples to calculate a DEM grid surface at a spatial resolution of 1 km. The remaining component comprised a test group that was used to analyse the DEM grid surface upscaling difference.

Based on the cross-validation results (Tables 6–8), the data show that HASM-US simulations encompass a mean difference of –2.32 m, an RMS difference of 19.49 m, and a mean absolute difference of 9.52 m. In contrast, the corresponding differences of the results from the other two kinds of conventional resampling are approximately −6.90 m, 28 m, and 15 m, respectively. The accuracy of the coarse-resolution DEM can be effectively improved by introducing sample data and performing DEM upscaling using the HASM-US algorithm. As shown in Tables 6–8, the mean difference is greater than zero, and it is therefore the case that the elevation values of the upscaling results in each method are slightly higher than the measured values.

**Table 6:** The cross-validation results of the HASM-US method (m).

No.	Maximum positive difference	Maximum negative difference	Mean difference	RMS difference	Mean absolute value difference
1	70.8652	−67.4786	−3.0737	21.2032	11.1320
2	102.7711	−64.7729	−2.4098	19.3822	8.7271
3	69.5070	−61.1456	−1.0749	20.5966	10.1077
4	28.0619	−53.7034	−3.4244	14.1766	7.1011
5	81.2707	−100.8970	−4.2955	28.5925	13.6596
6	47.8110	−76.1785	0.3276	17.0450	7.7831
7	36.5143	−101.1641	−3.0571	19.1792	9.0494
8	41.0946	−52.7582	−2.0311	14.7859	7.8049
9	8.4557	−58.7312	−5.3511	15.8967	7.2584
10	101.0216	−63.5454	1.1496	24.0901	12.5647

**Table 7:** The cross-validation results of bilinear interpolation method (m).

No.	Maximum positive difference	Maximum negative difference	Mean difference	RMS difference	Mean absolute value difference
1	151.0580	−159.1950	−10.4107	39.7168	21.7726
2	13.7890	−210.8130	−9.5805	37.4261	14.7424
3	21.2690	−147.2430	−6.9228	25.3250	12.0878
4	16.1540	−46.5140	−6.8215	16.4976	11.0165
5	19.5500	−78.4060	−3.8371	17.7881	11.4303
6	24.1110	−79.3320	−9.8015	23.5983	14.3620
7	29.7300	−64.0890	−6.5110	21.4298	14.0196
8	146.4140	−130.7300	−7.6593	32.3447	17.2825
9	151.2480	−55.9400	0.6156	26.0038	13.0000
10	143.8650	−94.7360	−7.9765	35.2007	20.8328

**Table 8:** The cross-validation results of nearest neighbor interpolation method (m).

No.	Maximum positive difference	Maximum negative difference	Mean difference	RMS difference	Mean absolute value difference
1	159.0580	−156.1950	−10.1071	40.1035	22.1993
2	14.7890	−230.8130	−9.3663	39.1058	14.9407
3	25.4400	−147.2430	−5.8692	24.6933	12.0479
4	22.1540	−60.5140	−8.1965	18.9082	12.8274
5	26.6180	−93.4060	−3.9264	19.0679	11.8114
6	29.1110	−85.8760	−9.4265	24.0209	14.4654
7	29.7300	−68.0890	−6.5824	21.6232	14.5196
8	154.4140	−134.7300	−8.4271	34.3522	18.5549
9	159.2480	−55.9400	0.7585	26.6980	12.8139
10	151.8650	−99.4760	−7.8731	36.4198	21.8471

**Figure 5:**
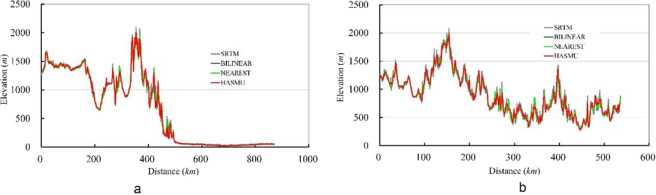
Elevation sections lines (notes: a and b express the elevation sections lines extracted in the northern region and western region of the BTH area, respectively).

In addition to cross validation, this study also qualitatively evaluated the effects of DEM upscaling simulations using cross-section line analysis. Thus, two elevation section lines were extracted in the northern and western regions of the BTH area (Fig. 5). Section lines of the relief were revealed as profiles by SRTM 4 DEM data, including the 1 km spatial resolution DEM profile obtained by bilinear interpolation, the nearest neighbour interpolation method, and the HASM-US method. The simulation results of HASM-US can therefore better capture extreme DEM points on the basis of section lines.

### Geomorphological Regionalization in the BTH Area

**Figure 6:**
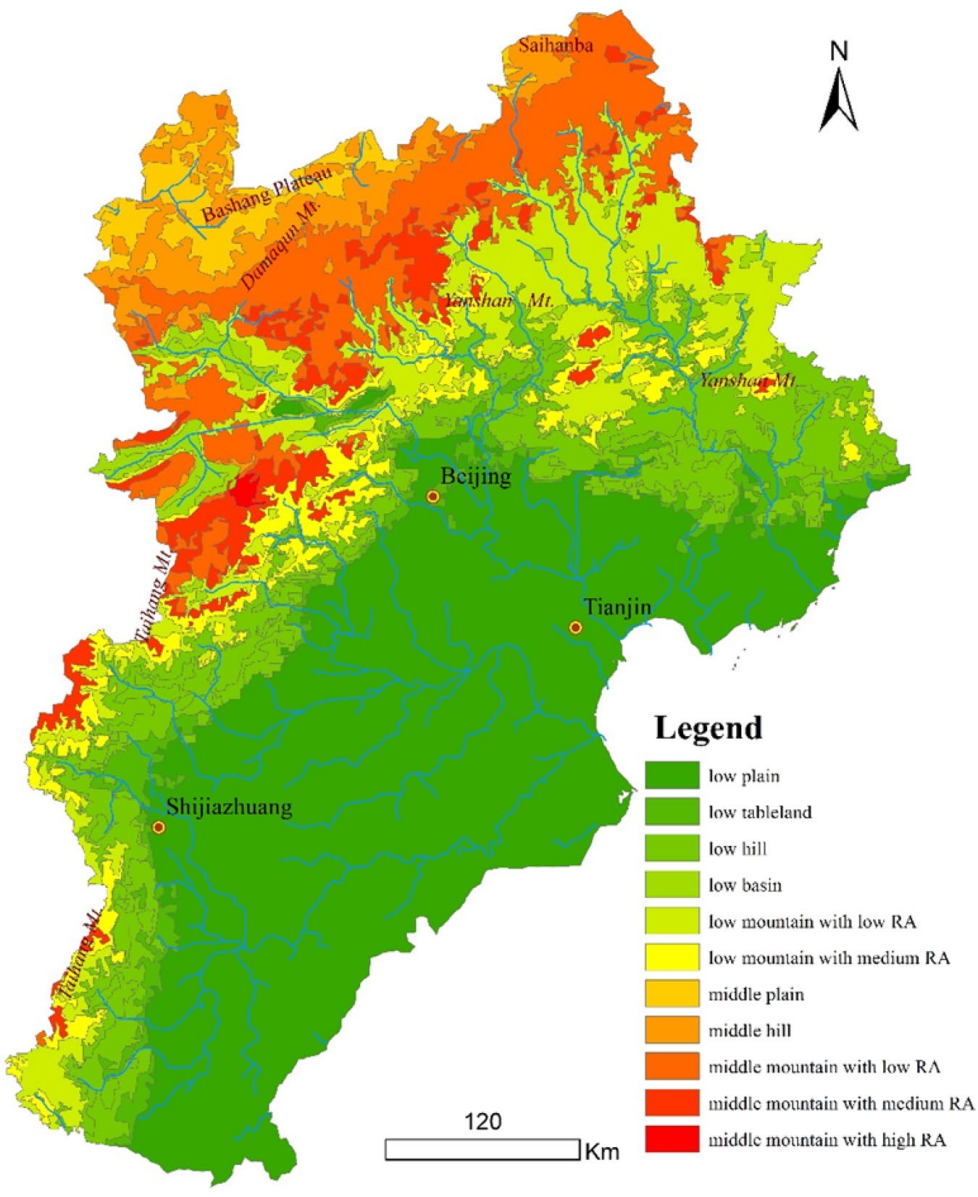
The distribution of geomorphological types in the BTH area.

Geomorphological types within low-altitude areas (<1,000 m) include six categories (Table 9, Fig. 6). The low plain area within BTH comprises approximately 8.98 × 10^4^ km^2^, 41.75% of the total area. The eastern and southern regions extend to the southeastern province boundary as well as to the coastline of Hebei Province, west to the eastern fringes of the Taihang Mountains, and north to the southern fringes of the Yanshan Mountains. On the western and northern sides of this region, the extent of this line is consistent with numerous highways and national highways and encompasses multiple towns and cities. The low-altitude tableland area within the BTH area encompasses 6,773 km^2^, 3.32% of the study area. These low hills are widely distributed and encompass a total area of approximately 3.20 × 10^4^ km^2^, 14.89% of the total area. The spatial pattern of this land type is a belt: the frontal mountain transition zone to the east of the Taihang Mountains as well as the transition zone to the south of the frontal Yanshan Mountains encompass areas of approximately 1.55 × 10^4^ km^2^ and 1.65 × 10^4^ km^2^, respectively. Low basins within the region encompass an area of 4,273 km^2^, 1.99% of the total area. The low mountain area with low RA values is 2.52 × 10^4^ km^2^, 11.70% of the total area, and includes the Taihang Mountain area south of Xiaowutai Mountain. This land area comprises a discontinuous band and is more continuous in the northeast, accounting for a large proportion of the Yanshan Mountain area. Low mountains with medium RA areas encompass approximately 9,884 km^2^, 4.59% of the total area.

**Table 9:** The areas of every geomorphological type in the BTH area.

Geomorphological types	Area/km^2^	Percentage
Low basin	4273.319	1.99%
Low hill	32041.92	14.89%
Low mountain with low RA	25188.21	11.70%
Low mountain with medium RA	9884.535	4.59%
Low plain	89844.97	41.75%
Low tableland	7154.535	3.32%
Middle plain	5497.618	2.55%
Middle hill	9841.989	4.57%
Middle mountain with high RA	263.133	0.12%
Middle mountain with low RA	22132.18	10.28%
Middle mountain with medium RA	9085.586	4.22%
total	215208	100.00%

The middle plain and middle hill regions comprise the main body of the Bashang Plateau. The first of these two regions comprises an area of 5,497 km^2^ (2.55%), while the latter region covers an area of 9,481 km^2^ (4.57%). The middle hill regions within the BTH area are mainly located in places such as the transition zone of Damaqun Mountain as well as on the middle plain. The middle mountain area with low RA values also comprises 2.21 × 10^4^ km^2^, 10.28% of the area. The middle mountain area within the BTH area that has medium RA values comprises 9,085 km^2^, 4.22% of the total area. This kind of land is widely distributed but scattered, including on the eastern side of the Taihang Mountains, the eastern part of Xiaowutai Mountain, and in the middle-western region of the Yanshan Mountains. The area of middle mountain land with high RA values comprises approximately 263 km^2^, 0.12% of the total area.

### Geomorphological Features Analysis in the BTH Area

Geomorphological patterns within the BTH region reveal a basic framework that comprises more low plains in the southeast as well as more mountainous areas in the west and north. The southern section of the western part of the study area is rapidly decreasing in altitude from the Taihang Mountains to the east onto low plains. The profile curve for the BTH area is therefore jagged and is characterized by an obvious downward trend. Low-altitude hills in the mountain fronts are wide; mountain basins and high-amplitude mountains interlace with one another and are distributed in the northern section of the western part of the BTH area. Subsequent to the highest Xiaowutai Mountain peak, landforms rapidly decrease to a low plain, while the low hilly area in front of the mountain is narrow. Moving from the middle plains and hilly areas on Bashang in the northwest to the southeast, Damaqun Mountain and other areas increase in amplitude before gradually falling to low plains, while wide valleys develop between mountains. The relief of the Yanshan Mountain district in the north generally decreases from north to south; the profile line across this area is extremely tortuous and indicates strong tectonic movement across this area. The terrain is also relatively fragmented within this region because of river erosion.

## Discussion

### DEM Upscaling methods

Upscaling conversion of DEM data is necessary before they are used to join the simulation and analysis with other data at different scales. The methods of upscaling DEM data mainly include classical resampling methods, terrain simplification based on the LOD model^32^, the Douglas Peucker algorithm^33^, the point spread function^34^, the filtering method^35^, wavelet transform^36^, fractal^37^, etc., which are mainly used for simplification of the terrain information of scattered points; some are mainly used for DEM scale conversion of TIN format, but the most widely used method is resampling.

The general resampling method (e.g., the nearest neighbour sampling and bilinear interpolation methods), as a grid data processing method, is the most classical method of upscaling transformation of grid data, which is supported by mainstream GIS software and widely used in remote sensing image processing and DTM spatial analysis^38,39^. The accuracy of the DEM will decrease with increasing grid resolution when the DEM data are upscaled, and different resampling methods will have different effects on the accuracy of upscaled DTM data^40^.

The measurement accuracy, resolution of DEM data, terrain relief, pixel size, and modelling error of DEM model directly affect the accuracy of DEM data^20,41^. On the same condition of the measurement accuracy, terrain complex level and resolution of DEM data, the modelling error of DEM is mainly decided by the adopted upscaling model. However, in order to reduce the amount of data and maintain more terrain details in the upscaled DEM data, some elevation samples are usually added to participate in the upscaling transformation of the DEM in the classical upscaling methods^42–44^. Moreover, the resampling method, wavelet transform, fractal and other classical methods can’t deal with upscaling the DEM data when some new sample data are joined for simulation. The HASM-US method developed in this paper can not only involve these new sample data in the upscaling process of DEM but also decrease the accuracy loss of the upscaled DEM data and retain more terrain details in the original DEM data

### Geomorphological patterns in the BTH Area

Terrain is one of the most fundamental components of the natural environment^31,45^. Geomorphological regionalization is key to the study of the spatial differentiation of geographical phenomena^31^. The similarities and differences of the combination of landform types and the causes of landforms are the basis of geomorphological zones at all levels^31^. There are various methods for the classification of landform types, but they have similar characteristics in general. Based on remote sensing and GIS methods, Boccao (2001)^46^ used the parameters of relief amplitude, slope steepness and dominant lithology to classify major landforms. Gao (2019)^47^ made geomorphological divisions of China on the basis of regional differences and the formation of landforms. Liu (2016)^48^ divided the landform zones of the North China Plain according to morphology and formation. Cheng (2017)^49^ characterized the geomorphological zones on the northwestern edge of the Qinghai-Tibet Plateau with topographic profiles and gradients based on digital elevation model (DEM) data. With remote sensing images and SRTM-DEM and other multisource data, intelligent regionalization can be realized using the methods of geographical grid and systematic cluster analysis^50^. Therefore, with the development of remote sensing and geographic information technology, rapid regionalization can be achieved based on digital terrain analysis (DTA) of DEMs. Regarding the geomorphological regionalization of the BTH area, Zhao and Cheng (2016) first divided the landform types into plains and mountains based on remote sensing images, DEMs and historical geomorphological maps and then calculated the relief amplitude of the SRTM-DEM and classified it as plains, tablelands, hills, and mountains with low, medium and high relief amplitudes^51^. In this paper, 2 primary parameters were selected from 7 topographic parameters based on the DTA of more accurate DEMs and PCA and then classified as the quantitative criterion for geomorphological regionalization, in which the subjectivity can be reduced to the greatest extent. Compared to Zhao's program, the grading of elevation and subdivisions of plains and hills produced 11 types; the diverse geomorphological regions in our study area can characterize the topographic features in detail.

## Conclusions

Multisource elevation data were upscaled in this analysis to obtain DEM grid data with a resolution of 1 km based on the HASM-US method; seven geomorphological parameters, including *EL*, *SL*, *SA*, *RA*, *SI*, *SR*, and *PC*, were calculated using ArcGIS software. Dominant geomorphological factors were extracted via PCA for geomorphological classification. This analysis leads to a number of key conclusions.

First, cross validation and surface profile analyses reveal that HASM-US enables higher accuracy and better reflects extreme values within DEMs in terms of upscaling simulation results when compared to those of either bilinear interpolation or nearest neighbour interpolation methods. HASM-US is therefore a more efficient method for upscaling DEMs.

The application of PCA reveals that elevation and *RA* provide representative factors for characterizing regional geomorphological features. Thus, combining these two, the BTH study area can be divided into 11 geomorphological types: low plains, low tablelands, low hills, low basins, low altitude mountains with low *RA* values, low altitude mountains with medium *RA* values, middle plains, middle hills, middle altitude mountains with low *RA* values, middle altitude mountains with medium *RA* values, and middle altitude mountains with high *RA* values. Low plains are the major land type within the BTH region and account for 40.58% of the total area. Trends in amplitude changes vary greatly along east–west, north–south, and northwest–southeast transect directions.
